# Association of Blood Pressure Variability and Intima-Media Thickness With White Matter Hyperintensities in Hypertensive Patients

**DOI:** 10.3389/fnagi.2019.00192

**Published:** 2019-08-06

**Authors:** Xin Chen, Yingqian Zhu, Shasha Geng, Qingqing Li, Hua Jiang

**Affiliations:** Department of Geriatrics, Shanghai East Hospital, Tongji University School of Medicine, Shanghai, China

**Keywords:** white matter hyperintensities, carotid intima-media thickness, ambulatory blood pressure, ambulatory blood pressure variability, hypertension

## Abstract

**Background and Purpose**: Ambulatory blood pressure variability (ABPV), ABP, and carotid intima-media thickness (IMT) are closely associated with white matter hyperintensities (WMH), and few studies focused on establishing effective models based on ABP, ABPV, and IMT to predict the WMH burden. We aimed to evaluate the value of a predictive model based on the metrics of ABP, ABPV, and IMT, which were independently associated with the WMH burden.

**Methods**: We retrospectively enrolled 140 hypertensive inpatients for physical examinations in Shanghai East Hospital, Tongji University School of Medicine between February 2018 and January 2019. The basic clinical information of all subjects was recorded, and we also collected the metrics of ABP, ABPV, and IMT. Patients with Fazekas scale grade ≥2 were classified into heavy burden of WMH group. Then, we analyzed the association between all characteristics and the WMH burden. Multivariate analysis was performed to assess whether the metrics of ABP, ABPV, and IMT were independently associated with WMH, and we used receiver operating characteristic (ROC) to evaluate the value of predictive model based on the metrics of ABP, ABPV, and IMT.

**Results**: Higher WMH grade was associated with increasing age, diabetes mellitus, higher total cholesterol (TC), higher low-density lipoprotein (LDL), higher IMT, higher 24-h systolic blood pressure (SBP), higher daytime SBP, higher nocturnal SBP, 24-h and daytime standard deviation (SD) of SBP, and 24-h SBP weight SD; 24-h SBP, 24-h SBP-SD, and IMT were independently related to the burden of WMH even after adjusting for the clinical variables. In addition, we also established a model that has a higher predictive capacity using 24-h SBP, 24-h SBP-SD, and IMT in the ROC analysis to assess the WMH burden in hypertensive patients.

**Conclusions**: Higher 24-h SBP, higher 24-h SBP-SD, and larger IMT were independently associated with a greater burden of WMH among elderly primary hypertension Asian patients. Establishing a model based on these factors might provide a new approach for enhancing the accuracy of diagnosis of WMH using metrics in 24-h ABPM and carotid ultrasound.

## Introduction

White matter hyperintensities (WMH), also named white matter lesions, are commonly observed in the elderly, usually detected on magnetic resonance imaging (MRI) with hyperintense signal appearances on T2-weighted MRI (Fazekas et al., 1993). WMH are considered as a manifestation of cerebral small vessel disease (CSVD; Wardlaw et al., 2013), and its extensive lesions are highly related to the risk of cognitive decline (Altamura et al., 2016), stroke (Fazekas et al., 1993; Jeerakathil et al., 2004), and gait disturbance (Polvikoski et al., 2010). As the pathogenesis of WMH has not been completely understood, it might be in the subclinical stage for long before the onset of the first clinical manifestations (Pantoni, 2010). Therefore, early detection of patients with subclinical CSVD may be effective in preventing future adverse prognosis.

WMH have been identified to be more prevalent in people with hypertension (Jiménez-Balado et al., 2019). Twenty-four-hour ambulatory blood pressure (ABP) monitoring (24-h ABPM) has been conformed as a more scientific method to predict blood pressure-related brain damage than office blood pressure measurement (Ohkubo et al., 2000). Previous studies have proven that increased 24-h ABP variability (ABPV) and higher 24-h ABP levels are closely associated with WMH (Yamaguchi et al., 2014; Filomena et al., 2015). In addition, subclinical atherosclerotic changes have been reported to be related to CSVD and WMH burden (Rundek et al., 2017). Intima-media thickness (IMT) is not only an effective marker of subclinical atherosclerosis but also a predictive factor of cardiocerebrovascular disease (Lorenz et al., 2007). A recent study has shown that increased IMT is independently associated with a heavier burden of WMH among elderly and Hispanic people (Della-Morte et al., 2018). However, to our knowledge, only a few studies focused on establishing effective models based on ABP, ABPV, and IMT to predict the burden of WMH among Asian primary hypertension patients. In this study, we investigated the association of the ABP, ABPV, and IMT metrics with the burden of WMH, Moreover, we also evaluated the value of predictive model based on the metrics of ABP, ABPV, and IMT, which were independently associated with the WMH burden.

## Materials and Methods

### Participants

This was a retrospective, cross-sectional study. We recorded information of 140 hypertensive inpatients for physical examinations due to headache or dizziness in Shanghai East Hospital, Tongji University School of Medicine between February 2018 and January 2019. Hypertension was defined as systolic blood pressure (SBP) ≥140 mm Hg and/or diastolic blood pressure (DBP) ≥90 mmHg. The inclusion criteria were: (1) patients with primary hypertension diagnosed ≥1 year; (2) age ≥40 years old; and (3) underwent 24-h ABPM, carotid IMT (cIMT) measurements, and MRI scan within 30 days. The exclusion conditions were: (1) with secondary hypertension; (2) with history of stroke or dementia; (3) with large-vessel cerebrovascular diseases; and (4) with severe infections, severe nephrosis or liver diseases, or tumors. We recorded the basic information of all patients: age, sex, disease history, smoking history, body mass index, C-reactive protein (CRP), glucose, triglyceride (TG), total cholesterol (TC), high-density lipoprotein (HDL) cholesterol, and low-density lipoprotein (LDL) cholesterol. This study was approved by the Ethics Committee of Shanghai East Hospital, Tongji University School of Medicine.

### Twenty-Four-Hour ABPM

All participants underwent 24-h ABPM with an automated device (TM-2430, AND, Tokyo, Japan), which has been verified in accordance with the protocol of the British Hypertension Society (Palatini et al., 1998). Patients underwent ABPM during their hospital stay. They were asked to follow their usual activities without physical exercise or excessive movement on the non-dominant arm during ABPM recordings. Blood pressure was measured every 30 min between 6:00 am and 10:00 pm (day time), and every 60 min between 10:00 pm and 6:00 am (night time). We recorded mean SBP, mean DBP, blood pressure variability (BPV) [which included average real variability (ARV), coefficient of variation (CV), and weighted standard deviation (SD) of SBP and DBP].

ARV was defined as the absolute differences of consecutive measurements and was calculated according to the following formula: $\text{ARV = 1/}\sum w\times \sum_{k=1}^{N-1}w\times |BP_{k+1}-BP_{k}|$ (Cretu et al., 2016). CV was defined as the ratio between the SD and the mean SBP or DBP at the same period and was calculated by the following formula: CV = SD/mean BP (Bilo et al., 2007; Filomena et al., 2015). Weighted SD (wSD) was defined as the mean of day and night SD values corrected for the number of hours included in each of these periods, it was calculated by the following formula: wSD = (SD_day time_ × *T*_day time_ + SD_night time_ × *T*_night time_)/*T*_day time+night time_ (Bilo et al., 2007; Filomena et al., 2015). In addition, we also calculated nocturnal systolic dip status according to the following formula: [(SBP_day time_ − SBP_night time_)/SBP_day time_] × 100% to assess the circadian variation, and normal status was considered as the value between 10% and 20% dip (O’Brien et al., 2013).

### Carotid Ultrasound

Carotid ultrasound was performed in accordance with the standard scanning and reading protocols by a well-trained physician. cIMT was automatedly measured using a high-resolution B-mode ultrasound system (SSA-250A, Toshiba, Tokyo, Japan), which can improve precision and reduce variance of the measurements. We scanned arteries to visualize the IMT on the posterior or distal wall of the artery, and the measurements were made outside the areas of plaque (Touboul et al., 2007). All measurements were performed on frozen images. The two best-quality images were selected for analysis of each artery. IMT was defined as the distance from the anterior margin of the first echogenic line to the anterior margin of the second line. The first line represents the intima–lumen interface, and the second line represents the collagen-containing top layer of adventitia. All IMT values were calculated as the average of six measurements. We defined the carotid artery segments like the Northern Manhattan Study (Della-Morte et al., 2018): (1) near and far wall of the segment extending from 10 to 20 mm proximal to the tip of the flow divider into the common carotid artery; (2) near and far wall of the carotid bifurcation beginning at the tip of the flow divider and extending 10 mm proximal to the flow divider tip; and (3) near and far wall of the proximal 10 mm of the internal carotid artery. We recorded the mean cIMT by calculating the means of the near and far wall IMT of all carotid segments (Rundek et al., 2002; Della-Morte et al., 2018; Koç and Sümbül, 2019). These were previously reported with excellent reliability in the Northern Manhattan Study (Della-Morte et al., 2018).

### MRI Data and Measurement of WMH

Brain MRI was measured using 1.5-Tesla MRI (Philips Medical Systems, Best, Netherlands), which included diffusion-weighted, T1-weighted, and T2-weighted imaging, fluid-attenuated inversion recovery (FLAIR), and susceptibility-weighted imaging (SWI). The sections were 5 mm thick. WMH were rated in FLAIR sequences in accordance with the Fazekas scale. Imaging markers of WMH were defined as follows: for periventricular: grade 0 (absent lesions), grade 1 (caps or pencilthin lining), 2 grade (smooth halo), and grade 3 (irregular periventricular lesions extending into the deep white matter); and for deep white matter: grade 0 (absent), grade 1 (punctuate foci), grade 2 (beginning of confluent foci), and grade 3 (large confluent areas; Fazekas et al., 1987). We classified patients into heavy burden of WMH group when their grade is ≥2 in either the periventricular or in the deep white matter according to the Fazekas scale. All MRI examinations were independently assessed by two experienced neurologists who were blind to other clinical variables. In case of disagreement, lesions were ascertained by consensus. An intrarater reliability test was performed in 140 subjects. Interreader- and intrareader-intraclass correlation coefficients for periventricular WMH scores were 0.88 and 0.83, respectively. In addition, the interreader- and intrareader-intraclass correlation coefficients for subcortical WMH scores were 0.86 and 0.89, respectively. Pearson’s correlation coefficient between periventricular and subcortical WMHs was 0.74.

### Statistical Analysis

The statistical analyses were performed using SPSS22.0 software (IBM SPSS, Armonk, NY, USA). The categorical variables of clinical features were expressed as number and percentage and analyzed with the chi-square test. The Mann–Whitney *U*-test or Student’s *t*-test was used to comparing continuous variables of clinical characteristics, which were expressed as mean ± SD. Potential risk markers and variables for which the *P* < 0.05 in univariate analysis were included in the multivariate logistic regression analysis. Forward elimination multivariate logistic regression analyses were performed. Parameters of ABP, ABPV, and IMT with *P* < 0.05 were included in receiver operating characteristic (ROC) analysis to show their evaluated values and establish a combination. *P* < 0.05 was considered to be statistically significant.

## Results

A total of 140 patients were enrolled in this study. The average age was 69.24 ± 10.54 years, and 54.29% were male. Seventy-five percent of them were treated with BP-lowering agents (Table 1).

**Table 1:** Baseline factors associated with the burden of WMH.

Characteristics	All Subjects *n* = 140	0–1 grade *n* = 94	2–3 grade *n* = 46	*P*-value
Age (years)	69.24 ± 10.54	66.47 ± 10.12	74.89 ± 9.09	0.001*
Male (%)	76 (54.29%)	47 (50.00%)	29 (63.00%)	0.146
Diabetes mellitus (%)	51 (36.43%)	27 (28.72%)	24 (52.17%)	0.007*
Smoking habit (%)	37 (26.43%)	25 (26.60%)	12 (26.09%)	0.949
Central obesity (%)	98 (70.00%)	65 (69.15%)	33 (71.74%)	0.753
Body mass index	28.75 ± 4.22	28.71 ± 4.34	28.81 ± 4.26	0.831
CRP	4.96 ± 1.51	4.05 ± 1.57	6.73 ± 1.46	0.153
Glucose	5.74 ± 1.63	5.61 ± 1.51	6.02 ± 1.84	0.163
TG	1.69 ± 1.13	1.78 ± 1.24	1.49 ± 0.85	0.143
TC	4.68 ± 1.22	4.83 ± 1.20	4.38 ± 1.23	0.041*
HDL	1.15 ± 0.28	1.17 ± 0.28	1.13 ± 0.27	0.494
LDL	2.48 ± 0.91	2.60 ± 0.88	223 ± 0.93	0.025*
IMT (mm)	0.711 ± 0.17	0.689 ± 0.17	0.77 ± 0.19	0.026*
Duration of hypertension (year)	8.33 ± 4.15	8.12 ± 4.96	8.45 ± 4.38	0.325
Nocturnal dipping of SBP	0.043 ± 0.081	0.052 ± 0.074	0.025 ± 0.094	0.067
Dipper status (%)	33 (23.57%)	23 (24.47%)	10 (21.74)	0.721
24-h SBP (mmHg)	128.76 ± 17.47	126.56 ± 17.51	133.24 ± 16.68	0.033*
24-h DBP (mmHg)	73.46 ± 9.54	73.89 ± 9.77	72.59 ± 9.09	0.448
Daytime SBP (mmHg)	131.65 ± 16.28	129.90 ± 16.67	135.22 ± 15.02	0.070*
Daytime DBP (mmHg)	75.75 ± 9.94	76.39 ± 10.15	74.43 ± 9.46	0.275
Nocturnal SBP (mmHg)	126.10 ± 19.80	123.27 ± 19.42	131.89 ± 19.50	0.015*
Nocturnal DBP (mmHg)	71.30 ± 9.93	71.51 ± 10.11	70.87 ± 9.64	0.721
BP-lowing treatment (%)	105 (75.00%)	69 (73.40%)	36 (78.26%)	0.533
**No. of BP-lowing agents**				
No treatment (%)	35 (25.00%)	25 (26.60%)	10 (21.74%)	0.533
1 BP-lowing agent (%)	78 (55.71%)	53 (56.38%)	25 (54.35%)	0.820
≥2 BP-lowing agents (%)	26 (18.57%)	16 (17.02%)	10 (21.74%)	0.500
**Class of BP-lowing drugs**				
ACEIs (%)	5 (3.57%)	2 (2.13%)	3 (6.52%)	0.188
ARB (%)	44 (31.43%)	30 (31.91%)	15 (32.61%)	0.934
β-Blockers (%)	3 (2.14%)	2 (2.13%)	1 (2.17%)	0.986
Dihydropyridinic CCB (%)	71 (50.71%)	47 (48.96%)	23 (50.00%)	0.989
Npnloop diuretics (%)	10 (7.14%)	7 (7.45%)	3 (6.52%)	0.842

WMH, white matter hyperintensities; CRP, C-reactive protein, glucose; TG, triglyceride; TC, total cholesterol; HDL, high-density lipoprotein cholesterol; LDL, low-density lipoprotein cholesterol; IMT, intima-media thickness; SBP, systolic blood pressure; DBP, diastolic blood pressure; ACEI, indicate angiotensin-converting enzyme inhibitor; ARB, angiotensin receptor blockers; CCB, calcium-channel block, *P < 0.05.

According to the MRI images, 94 patients were classified into 0–1 WMH grades, and 46 patients were classified into 2–3 WMH grades. In the univariate analysis (Table 1), the burden of WMH was associated with increasing age, diabetes mellitus, higher CH, higher LDL, higher IMT, higher 24-h SBP, higher daytime SBP, and higher nocturnal SBP (all *p* < 0.05). We also calculated the metrics of BPV in all periods and found that 24-h and daytime SD of SBP, and 24-h SBP weight SD significantly increased in those with 2–3 WMH grades (all *p* < 0.05; Table 2).

**Table 2:** Association between ABPV measurements and the burden of WMH.

BPV Measures	All Subjects *n* = 140	0–1 grade *n* = 94	2–3 grade *n* = 46	*P*-value
**Systolic BP**				
24-h ARV	10.34 ± 3.27	9.97 ± 2.75	11.22 ± 2.31	0.064
Awake ARV	10.81 ± 3.11	10.61 ± 3.15	11.44 ± 3.78	0.102
Asleep ARV	9.98 ± 3.46	9.87 ± 3.28	11.10 ± 3.27	0.057
24-h SD	13.41 ± 3.31	12.87 ± 2.88	14.54 ± 3.85	0.005*
24-h wSD	12.21 ± 3.22	11.59 ± 2.94	13.23 ± 3.43	0.008*
Awake SD	12.41 ± 3.72	11.61 ± 3.20	14.04 ± 4.18	0.001*
Asleep SD	11.79 ± 3.69	11.67 ± 3.12	12.03 ± 4.07	0.589
24-h CV	10.56 ± 2.66	10.28 ± 2.32	11.13 ± 3.21	0.077
Awake CV	9.49 ± 2.87	9.01 ± 2.54	10.47 ± 3.27	0.104
Asleep CV	9.58 ± 3.15	9.68 ± 2.60	9.37 ± 4.06	0.582
**Diastolic BP**				
24-h ARV	7.13 ± 2.16	7.25 ± 1.87	7.02 ± 2.03	0.557
Awake ARV	7.78 ± 2.63	7.89 ± 2.21	7.64 ± 2.01	0.476
Asleep ARV	6.53 ± 2.59	6.71 ± 2.24	6.49 ± 2.51	0.318
24-h SD	9.36 ± 2.06	9.33 ± 1.99	9.44 ± 2.23	0.757
24-h wSD	8.79 ± 2.11	8.70 ± 2.17	8.83 ± 2.45	0.746
Awake SD	8.88 ± 2.24	8.71 ± 2.02	9.24 ± 2.01	0.186
Asleep SD	8.53 ± 2.68	8.71 ± 2.31	8.19 ± 3.31	0.288
24-h CV	12.88 ± 2.99	12.76 ± 2.90	13.11 ± 3.18	0.516
Awake CV	11.86 ± 3.15	11.55 ± 2.97	12.48 ± 3.42	0.099
Asleep CV	12.18 ± 4.12	12.38 ± 3.59	11.75 ± 5.07	0.401

ABPV, ambulatory blood pressure variability; WMH, white matter hyperintensities; BPV, blood pressure variability; BP, blood pressure; ARV, average real variability; CV, coefficient of variation; SD, standard deviation; wSD, weighted standard deviation, *P < 0.05.

We used multivariate analysis to assess whether IMT, SBP levels (24-h SBP, daytime SBP, and nocturnal SBP), and BPV metrics (24-h and daytime SD of SBP, 24-h SBP weight SD) were independently associated with 2–3 WMH grades after adjustment by clinical variables (age, sex, diabetes mellitus, TC, LDL cholesterol, use of anti-hypertensive treatment, and the parameters of ABP, ABPV, and IMT with *P* < 0.05 in univariate analysis). We found higher 24-h SBP [odds ratio (OR): 1.063, 95% CI: 1.033–1.135, *P* = 0.036], 24-h SBP-SD (OR: 1.280, 95% CI: 1.117–1.552, *P* = 0.014), and IMT (OR: 1.207, 95% CI: 1.135–1.492, *P* = 0.002) were independent predictors of heavy burden of WMH (Table 3).

**Table 3:** Association of 24-h SBP, 24-h SBP-SD, and IMT with WMH.

Characteristics	OR (95% CI)	*P*-value
24-h SBP (mmHg)	1.063 (1.033–1.135)	0.036*
24-h SBP-SD (mmHg)	1.280 (1.117–1.552)	0.014*
IMT (mm)	1.207 (1.135–1.492)	0.002*

Models are adjusted by age, sex, diabetes mellitus, total cholesterol, low-density lipoprotein cholesterol, use of antihypertensive treatment, and the parameters of ABP, ABPV and IMT with *P* < 0.05 in univariate analysis. SBP, systolic blood pressure; SD, standard deviation; IMT, intima-media thickness; WMH, white matter hyperintensities; OR, odd ratio; CI, confidence interval, *P < 0.05.

**Figure 1:**
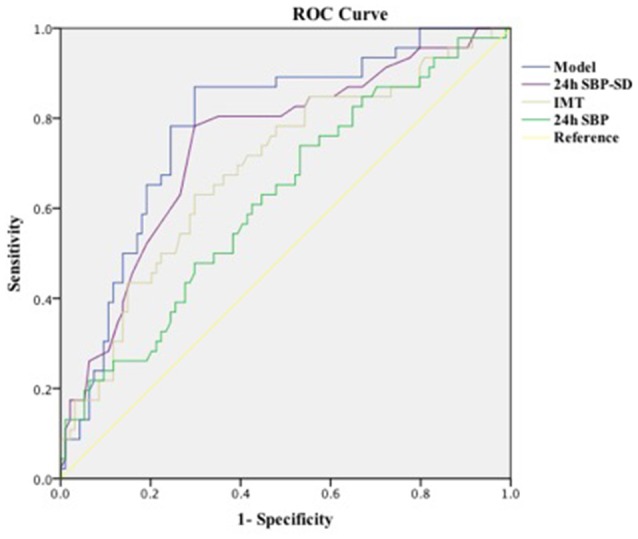
Curves of receiver operating characteristic.

We performed ROC analysis to show the predictive capacity of the 24-h SBP [area under curve (AUC): 0.688, 95% CI: 0.594–0.783, *P* = 0.020], 24-h SBP-SD (AUC: 0.742, 95% CI: 0.653–0.830, *P* = 0.001), and IMT (AUC: 0.711, 95% CI: 0.622–0.819, *P* = 0.001). We saved probabilities of 24-h SBP, 24-h SBP-SD, and IMT in multivariate analysis and obtained the predictive model (AUC: 0.785, 95% CI: 0.705–0.865, *P* = 0.001) in the ROC analysis; the AUC of the model was higher than any of the 24-h SBP, 24-h SBP-SD, and IMT (Table 4; Figure 1).

**Table 4:** Receiver operating characteristic model and the predictive values.

Characteristics	AUC (95% CI)	*P*-Value
24-h SBP (mmHg)	0.688 (0.594–0.783)	0.020*
24-h SBP-SD (mmHg)	0.742 (0.653–0.830)	0.001*
IMT (mm)	0.711 (0.622–0.819)	0.001*
Model	0.785 (0.705–0.865)	0.001*

*AUC, Area Under Curve; CI, confidence interval; SBP, systolic blood pressure; SD, standard deviation; IMT, intima-media thickness*, *P < 0.05.

## Discussion

The major findings in our study were that: (1) higher WMH grade was associated with increasing age, diabetes mellitus, higher TC, higher LDL, higher IMT, higher 24-h SBP, higher daytime SBP, higher nocturnal SBP, 24-h and daytime SD of SBP, and 24-h SBP weight SD; and (2) 24-h SBP, 24-h SBP-SD, and IMT independently related to the burden of WMH even after adjusting for the clinical variables. (3) In addition, we established a model with a higher predictive capacity using 24-h SBP, 24-h SBP-SD, and IMT in the ROC analysis to assess the WMH burden in hypertensive patients.

In this study, we found higher 24-h SBP level was the only ABP metric that was independently associated with the higher grade of WMH (2–3 grades). This result was consistent with previous studies suggesting the necessity of measurement of ABP among hypertensive patients for predicting the burden of WMH (Stewart et al., 2009; Filomena et al., 2015). The Rotterdam study showed that not only higher SBP but also higher DBP were independent predictive factors of the progression of periventricular WM disease (van Dijk et al., 2008). In a recent study, increased DBP was demonstrated to be associated with the brain WMH score, but the role of SBP was less clear (McNeil et al., 2018). Similarly, another study also found no significant relationship between SBP and WMH, whereas the 2-year change from baseline to year 2 in ambulatory SBP predicted the 2-year WMH change (Wolfson et al., 2013). The differences in results might due to the different included criteria and different ethnicity, but all these results emphasize the essentiality of considering individual different BP components with regard to WMH. In this study, we found 24-h SBP-SD was the only independent factor that related to the burden of WMH among all the ABPV metrics, and consistent findings could be found in several other studies (Wardlaw et al., 2013; Yamaguchi et al., 2014; Yang et al., 2018). However, 24-h SBP-wSD was not independently associated with the burden of WMH after adjusting all clinical variables; this may be caused by the sample size and inclusion criteria. In addition, a Chinese study found that SD and CV of SBP, CV of DBP in 24 h, daytime and nighttime and SD of DBP in nighttime were positively associated with the degree of enlarged perivascular spaces (EPVSs), which was closely related to WMH (Yang et al., 2017a,b). In the same year, this team also indicated that higher SBP levels were independently associated with EPVSs in basal ganglia but not in center semioval, which supported EPVSs to be a marker of CSVD (Yang et al., 2017a,b). In another study, higher variability in SBP that was self-measured at home (HBP) was also shown to be related to the progression of brain WMH (Liu et al., 2016). In contrast, Filomena et al. (2015) found that among all the ABPV metrics, short-term ARV of SBP was independently related to the presence of CSVD, but not SD of SBP. The different results might be attributed to the differences in scoring methods.

The underlying pathologic mechanisms of the association between BP and ABPV levels and WMH burden have not been fully understood. Increased permeability of the small vessel walls and damage of the blood brain barrier (BBB) have been demonstrated to contribute to the development of WMH. A previous study found that contrast agents leaked much more in the area of perforating arterial in patients with WMH than in normal people (Starr et al., 2003). Another study used the ratio of CSF and serum albumin to show BBB permeability and discovered that the burden of WMH was associated with the permeability of BBB (Wallin et al., 1990). Increased BP levels and ABPV would cause more stress on vessel walls, which might further cause endothelial injuries and arterial stiffness (Schillaci et al., 2012; Diaz et al., 2013). Thus, it is likely that higher levels of BP and ABPV could lead to the development of WMH through endothelial injuries. Furthermore, WMH was thought to originate from ischemic injury. Yao et al. (1992) found that ischemia in white matter regions could be observed by the increased proportion of oxygen uptake in those regions. In recent studies, researchers found that changes in hemodynamics might contribute to the ischemia of white matter regions (Mok et al., 2012; Poels et al., 2012). The impairment of cerebral blood flow (CBF) has been considered as the most common type of hemodynamic change. Increased ABPV levels with sudden changes in BP might lead to cerebral hypoperfusion and development of WMH. Moreover, WMH could contribute to higher 24-h ABPV. The results in this study showed that 24-h SBP and 24-h SBP-SD, but not that of DBP, were independently related to the burden of WMH. Further studies are still needed to explore the underlying mechanisms.

In this study, we also reported a significant association between IMT and WMH. cIMT has been reported to be closely related to brain MRI changes (Pico et al., 2002). In a cardiovascular health study, researchers found increased IMT was strongly associated with WMH (Manolio et al., 1999). Similarly, other researchers reported in elderly hypertensive patients with memory disorder, in elderly patients with Alzheimer’s disease (AD), or vascular dementia patients a significant association between IMT and leukoaraiosis on MRI could be observed (Kearney-Schwartz et al., 2009; Altamura et al., 2016). In addition, increased IMT has been demonstrated as a risk factor of lacunar infarction, and lacunar infarction might result in increased WMH grade (Manolio et al., 1999; Tsivgoulis et al., 2005). These findings were consistent with the results in our study, and all these results revealed that increased IMT might be a useful marker of WMH.

Even though the exact pathophysiological changes are still unclear, some molecular mechanisms have been proposed as the link between IMT and WMH. In a postmortem study, WMH was found to be related to the impairment of arterioles (e.g., cellular wall thickening), revealing that cerebral arteriosclerosis might be one of the important factors in the development of WMH (Pantoni and Garcia, 1995). Moreover, changes in the large arterial wall might alter the cerebral microcirculation and lead to chronic brain hypoxia, which in turn contributes to the development of WMH. A previous study has indicated that the cerebral microcirculation was especially sensitive to increased pulsatile stress that might ultimately cause microvascular damage and WMH (Gutierrez et al., 2015). The molecular mechanisms of this process were likely mediated by the upregulation of proinflammatory and pro-growth factors leading to increased IMT Della-Morte and Rundek (2016). Furthermore, WMH could be caused by lower CBF, and lower CBF velocity, in turn, was associated with the increased IMT; atherosclerosis was considered to play a major role in this process (Appelman et al., 2008; Kwater et al., 2014).

To our best knowledge, although many studies have demonstrated ABPV, ABP, and IMT were closely associated with WMH (Filomena et al., 2015; Yang et al., 2017a,b), few studies established predictive model of the WMH burden using ABP level, ABPV level, and IMT. We established a model with a higher predictive capacity using 24-h SBP, 24-h SBP-SD, and IMT in ROC analysis to evaluate the WMH burden in patients with hypertension. This could enhance the accuracy of evaluating WMH burden in clinical application. Moreover, both 24-h ABPM and carotid ultrasound are noninvasive tests with easy operation and low cost. They can be effective in predicting the WMH burden in hypertensive patients. This study might provide a measuring method of discrimination of metrics in 24-h ABPM and carotid ultrasound to diagnose WMH.

This study has not only strengths but also limitations. As this was a retrospective, single-center, and small-scale study, this might cause higher selection biases. In addition, no classification of hypertension grade was recorded, which might influence the results. Moreover, the 24-h ABPM was performed during the hospital stay, and so the results of this study may not necessarily applicable to outpatients. Therefore, In the future, multicenter, prospective, and large-scale studies are still needed to clarify these problems.

In conclusion, results obtained from this study showed that 24-h SBP, 24-h SBP-SD, and IMT were independently associated with the WMH burden. Meanwhile, we established a model using 24-h SBP, 24-h SBP-SD, and IMT in ROC analysis to assess the WMH burden in patients with hypertension. This study might provide a new approach for enhancing the accuracy of diagnosis of WMH using metrics in 24-h ABPM and carotid ultrasound.

## Data Availability

The raw data supporting the conclusions of this manuscript will be made available by the authors, without undue reservation, to any qualified researcher.

## Author Contributions

HJ designed the study. XC collected information and wrote the article. YZ, SG, and QL reviewed and revised the article before the submission. They also made a great contribution to search literature during the process of Interactive Review. Moreover, they gave a lot of useful advices.

## Conflict of Interest Statement

The authors declare that the research was conducted in the absence of any commercial or financial relationships that could be construed as a potential conflict of interest.
